# Application of the Physical Disector Principle for Quantification of Dopaminergic Neuronal Loss in a Rat 6-Hydroxydopamine Nigral Lesion Model of Parkinson's Disease

**DOI:** 10.3389/fnana.2017.00109

**Published:** 2017-12-08

**Authors:** Katrine Fabricius, Pernille Barkholt, Jacob Jelsing, Henrik H. Hansen

**Affiliations:** Gubra, Hoersholm, Denmark

**Keywords:** physical disector, autodisector, stereology, 6-OHDA rat model, substantia nigra, Parkinson Disease

## Abstract

Stereological analysis is the optimal tool for quantitative assessment of brain morphological and cellular changes induced by neurotoxic lesions or treatment interventions. Stereological methods based on random sampling techniques yield unbiased estimates of particle counts within a defined volume, thereby providing a true quantitative estimate of the target cell population. Neurodegenerative diseases involve loss of specific neuron types, such as the midbrain tyrosine hydroxylase-positive dopamine neurons in Parkinson's disease and in animal models of nigrostriatal degeneration. Therefore, we applied an established automated physical disector principle in a fractionator design for efficient stereological quantitative analysis of tyrosine hydroxylase (TH)-positive dopamine neurons in the substantia nigra pars compacta of hemiparkinsonian rats with unilateral 6-hydroxydopamine (6-OHDA) lesions. We obtained reliable estimates of dopamine neuron numbers, and established the relationship between behavioral asymmetry and dopamine neuron loss on the lesioned side. In conclusion, the automated physical disector principle provided a useful and efficient tool for unbiased estimation of TH-positive neurons in rat midbrain, and should prove valuable for investigating neuroprotective strategies in 6-OHDA model of parkinsonism, while generalizing to other immunohistochemically-defined cell populations.

## Introduction

Application of basic principles of stereology can be applied to the task of estimating the total number of particles in a three-dimensional object; these particles can be neurons, glial cells, or organelles. The stereological technique now known as the physical disector (meaning “two-sections”) was first described in 1984 (Sterio, 1984), and has since become an established tool in quantitative neurobiology (e.g., Kristiansen and Nyengaard, 2012). We emphasize that stereological techniques are based on unbiased principles, which means that estimations, when obtained according to geometrically-defined rules, are “without systematic deviation from the true value.” For quantification of the number and volume of three-dimensional objects, e.g., neuron numbers and sizes, stereology offers a variety of probes that are geometric structures with mathematical properties designed for application in the tissue under investigation. More specifically, the *disector probe* is a three-dimensional physical or optical probe used to estimate the total number of particles. The physical disector procedure uses pairs of neighboring sections lying some distance (h) apart, which must be close enough to infer what lies between the two sections and thus insure that all particles are counted (Sterio, 1984; Gundersen et al., 1988).

**Figure 1:**
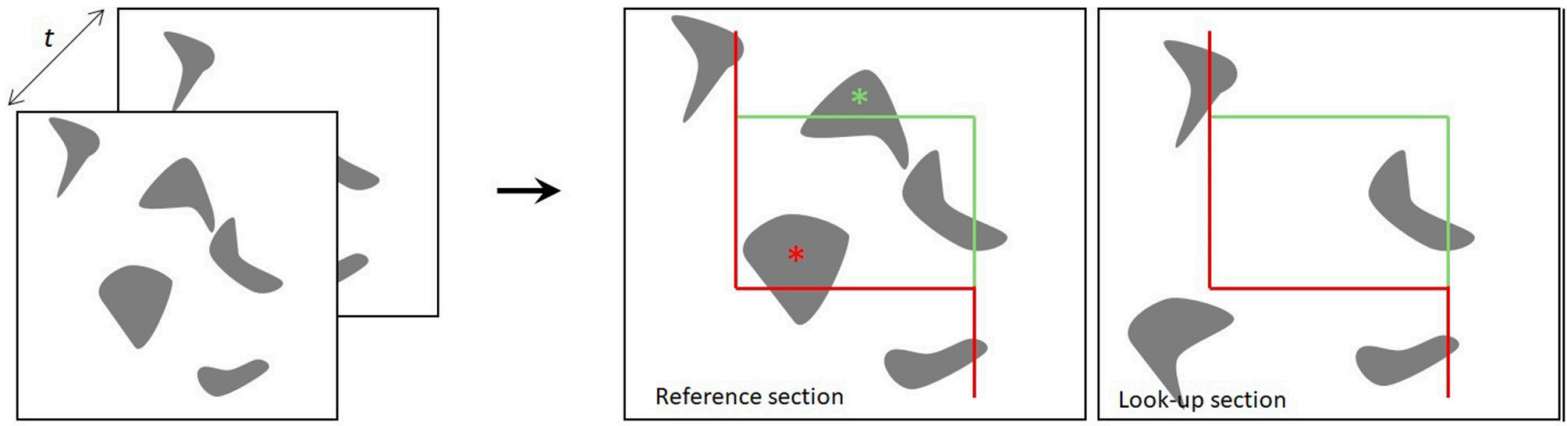
The principle of the physical disector. The distance t indicates the separation between the two sections. Two neighboring sections are superimposed, and the particles present within a counting frame in one section (reference section) but not the other (lookup section) are counted. The red lines of the counting frame are exclusion lines, and particles touching these boundaries are not counted. The green lines are inclusion lines (Gundersen, 1977; Gundersen et al., 1988).

The two sections for the physical disector are designated the reference section and the lookup section. If a particle appears in the reference section, but not in the lookup section, the particle is counted, such that each particle has an equal probability of being counted, irrespective of its size, shape, or orientation in the tissue (Gundersen et al., 1988; Figure 1). In applications of the optical disector, counting of particles requires moving the focal plane of the microscope in the z-axis at intervals within relatively thick tissue sections (e.g., 40 μm). Thus, a stack of thin focal planes is analyzed proceeding through the depth of the section. All unique identifiers (e.g., the nucleolus or nucleus of a neuron) within the disector height (h) are counted, after exclusion of guard zones above and below the disector, so as to avoid ambiguous identification or loss of cells at the end plane (West, 1999; Boyce et al., 2010; Tschanz et al., 2011). Here, the counting-frame consists of a square with one finite line (the inclusion line, green) and one infinite line (the exclusion lines, red lines) (Figure 1). The rule is that all particles inside or touching the unbiased counting frame are counted provided they do not touch the exclusion line. The frames are placed at equidistant X,Y-step within the region of interest on each section, and the volume is calculated knowing the frame area, h, and the magnification (Gundersen, 1977; Gundersen et al., 1988). Systematic uniform random sampling (SURS) gives a true representation of the whole population, providing that the first sampling step is chosen at random within the sampling frequency.

Recent years have seen increasing use of the stereological methods, notably for applications in the study of neurodegenerative diseases (Pakkenberg et al., 1991, 2003; Pakkenberg, 1993; Pakkenberg and Gundersen, 1997; Cabello et al., 2002; Stroeven and Hu, 2007; Eriksen et al., 2009; Boyce et al., 2010). Use of the physical disector can be rather time-consuming compared to the optical disector, due to the requirement that the experimenter must align the sections manually on a computer screen. This obstacle is circumvented by automated systems, which obtain an operator-independent alignment of microscopic images from the reference and lookup sections e.g., the Visiopharm newCast Autodisector system (Visiopharm, Denmark). Estimates can then be obtained off-line, with a considerable economy of effort due to the robust automatic alignment of images rather than manual alignment of tissue sections. The optical disector is often used for neuron counts, but the physical disector method is more suited for the thinner sections typically used in immunohistochemical staining protocols.

Parkinson's disease (PD) is a chronic and progressive neurodegenerative disorder with cardinal motor symptoms of tremor, rigidity and bradykinesia, which are largely attributed to the loss of dopamine (DA-ergic) neurons located in the *substantia nigra pars compacta* (SNc) of the mesencephalon (reviewed in Thomas and Flint Beal, 2007; Kalia and Lang, 2015). Animal models of PD have proved to be helpful in gaining an understanding of the underlying pathophysiology, and have also been instrumental in the discovery of novel treatments (e.g., Deumens et al., 2002; Eslamboli et al., 2003; Schober, 2004; Yuan et al., 2005; Blesa et al., 2012; Peoples et al., 2012; Torres and Dunnett, 2012). The classic PD model is obtained by infusion of the neurotoxin 6-hydroxydopamine (6-OHDA) to the medial forebrain bundle (MFB) or directly to SNc, which can induces a near ablation of the DA-ergic neurons (Jeon et al., 1995; Walsh et al., 2011). However, it is difficult in practice to obtain reproducible partial lesions of the nigrostriatal pathway with 6-OHDA infusions. The degeneration of nigrostriatal fibers leads to depletion of striatal dopamine content, but the associated effects on numbers of midbrain DA-ergic neurons are quantified in relatively few studies, generally without using stereological methods (e.g., Monville et al., 2006; Bertilsson et al., 2008; Torres and Dunnett, 2012; Hansen et al., 2016), although there are some stereological analyses in the 6-OHDA model (e.g., Petroske et al., 2001; Ma et al., 2009; Blesa et al., 2012; Peoples et al., 2012). We suppose that innovative stereological tools such as the autodisector and the use of digital slides can significantly simplify the procedure for quantitation of immunohistochemically-identified dopamine neurons (Keller et al., 2013). In this paper, we tested the applicability and efficiency of the physical disector design for the stereological evaluation of the number of midbrain TH-positive DA-ergic neurons in a conventional 6-OHDA model. Since results of this study have been presented elsewhere (Hansen et al., 2016), our present aim is to give a detailed technical account of the issues arising in the application of the autodisector and digital slides in a rat 6-OHDA model of PD. Others have previously reported on the effects of intoxication of Göttingen minipigs with methylenedioxymethamphetamine (MDMA) on the abundance of serotonin neurons of the dorsal raphe nucleus (Cumming et al., 2007). To our knowledge, the present 6-OHDA study is the first stereological study in a neurodegeneration model that combines the use of digital images of immunohistologically-stained brain sections using the automated aligning of images for the physical disector set-up.

## Methods and materials

### 6-OHDA lesion

All animal experiments were conducted in accordance with Gubra bioethical guidelines (internal ethics committee), which are fully compliant to internationally accepted principles for the care and use of laboratory animals. The described experiments were covered by personal licenses for Jacob Jelsing (2013-15-2934-00784) issued by the Danish Ethical Committee for animal research. A total of 40 male Sprague-Dawley rats (300–340 g, 7–8 weeks. Taconic DK) were used, in groups of 8–12 per treatment group. Four animals died during the study giving a total of 36 animals used for stereology. Rats were anesthetized with a mixture of 1.25 mg/ml midazolam, 2.5 mg/ml fluanisone and 0.079 mg/ml fentanyl (hypnorm/dormicum) at a dose of 2.7 ml/kg (s.c.). Animals were kept on a heating pad maintained on 36–37°C throughout surgery. A minimum of 20 min prior to 6-OHDA injection, animals were pre-treated with pargyline (a monoamine oxidase inhibitor; 5 mg/kg, i.p.) and desipramine (a noradrenaline uptake inhibitor; 25 mg/kg, i.p.) to prevent collateral damage to noradrenergic neurons. Animals were placed in a stereotaxic apparatus and (following sinus calibration for the lateral coordinates) received infusions of 6-OHDA (Cat# H116, Sigma- Aldrich, DK) in 0.01% ascorbic acid vehicle into the right medial forebrain bundle (MFB) using a Hamilton syringe with the tip placed at the following coordinates according to the atlas of Paxinos and Watson (1997); for partial nigral lesions (3 μg 6-OHDA in 2 μl), anterior-posterior (AP) −2.8 mm, lateral (L) −1.9 mm and ventral (V) −8.2 mm (relative to dura mater); full nigral lesion (total of 13.5 μg in 4.5 μl, delivered at two injection coordinates), AP = −4.4 mm L = 1.2 mm, DV = 7.8 mm (7.5 μg in 2.5 μl) and AP = −4.0 mm, L = 0.8 mm, DV = 8.0 mm (6 μg in 2 μl). The 6-OHDA infusions were made over 2 min, and the needle was left in place for another 5 min before being slowly retracted. Fresh 6-OHDA solutions were made from stock immediately prior to surgery and kept on ice until use. Intraoperative analgesia was obtained with carprofen (s.c., 0.1 ml/100 g body-weight of rimadyl 50 mg/ml, diluted 1:9 in isotonic NaCl) immediately prior to surgery. Rats received 4 ml NaCl i.p. post-surgically to restore fluid balance, and received daily post-surgical analgesia treatments with Baytril (5 mg/ml in 1 ml, s.c.) and Rimadyl (50 mg/ml in 1 ml) for at least 2 days post-surgery. Following the surgical procedures, all animals were monitored closely for signs of distress.

### Behavioral testing

R-(-)-Apomorphine hydrochloride hemihydrate (sigma-aldrich, DK Cat# A4393) and D-amphetamine (Sigma-aldrich, DK) induced rotations were assessed on weeks 1, 2, and 3 post-surgery. Rotations were counted using a Rotometer system (AccuScan Product Line, Omnitech Electronics Inc, USA). Following administration of r-(-)-apomorphine hydrochloride hemihydrate (0.05 mg/kg, s.c.) or D-amphetamine (5 mg/kg, s.c.), animals were placed in plastic bowls (diameter ~50 cm). The number of turns completed over a period of 15 min was counted, beginning at 10 min after apomorphine injection or 30 min after amphetamine injection, to accommodate the slower pharmacodynamics of amphetamine. Unilateral 6-OHDA-lesioned rats exhibit behavior super-sensitivity to dopaminergic agonists, which is reflected by amphetamine and apomorphine induced rotations (Ungerstedt and Arbuthnott, 1970), and is marked by upregulation of striatal dopamine D2/3 receptors on the lesioned side (Palner et al., 2011). The dopaminergic agonist apomorphine evokes contralateral turning, due to the direct stimulation of supersensitive dopamine D_2_-like and D_1_ receptors in the denervated striatum. In contrast, ipsiversive rotations is induced by indirect dopamine agonists such as amphetamine, due to their attenuated effect on dopamine release on the lesioned side (Ungerstedt and Arbuthnott, 1970; Hefti et al., 1980; Carman et al., 1991; Schwarting and Huston, 1996). Of the two agents, amphetamine is the stronger evoker of behavioral asymmetry. Thus, rats with more restricted lesions of the SNc do not show rotational asymmetry in response to apomorphine, such that animals with rotation exclusively after amphetamine administration exhibit a partial nigral lesion.

### Collection of brain tissue and storage

Animals were killed by decapitation while under acute CO_2_/O_2_ anesthesia. Brains were carefully removed and fixed by immersion in 4% parformaldehyde (PFA) for a minimum of 24 h at room temperature), followed by 48 h at t 4°C, whereupon brains were transferred to a 1% PFA solution and stored at 4°C until further processing.

### Collection of slides

**Figure 2:**
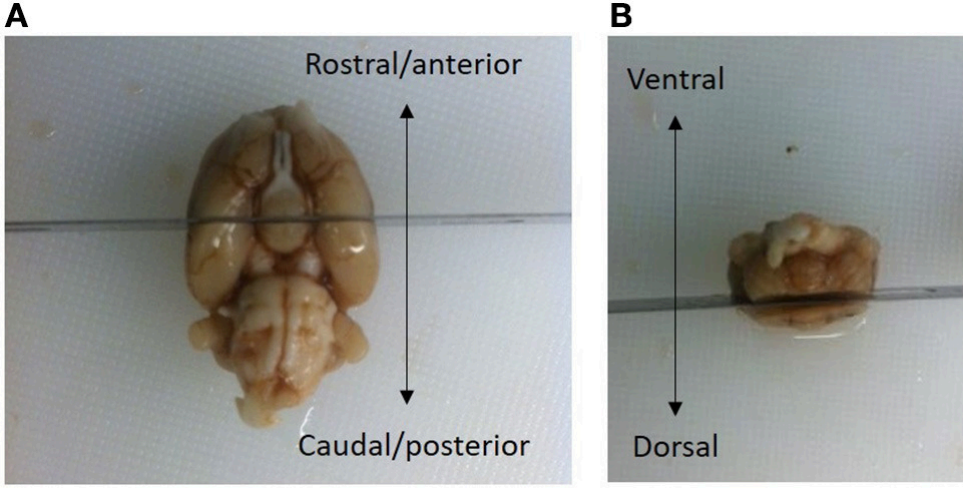
The brains were bisected in the coronal plane at a level posterior to the optic chiasm, i.e., −3.3 mm relative to bregma (Paxinos and Watson, 1997). **(A)** The superior cortical areas were trimmed away. **(B)** The brain in **(A)** is viewed from the ventral aspect, with the rosto-caudal axis indicated by an arrow. The brain in **(B)** is viewed in the caudal aspect with the dorsal/ventral axis indicated by an arrow. The frontal part of the cerebral hemispheres was discarded. A small vertical fiducial incision can be made in the top right brain hemisphere to confirm that the correct orientation at the time of sampling. After fixation, the part of the brain containing mesencephalon was weighed and embedded in pairs overnight in paraffin-blocks.

The brains were bisected in the coronal plane posterior of the optic chiasm at the level of bregma −3.3 mm according to the rat brain atlas of Paxinos and Watson (1997) (see Figure 2). The superior cortical areas were trimmed away. The caudal part was weighted (formalin weight) and paraffin-infiltrated overnight and then reweighed (paraffin weight). The difference between the wet formalin and paraffin weights is taken as an index of shrinkage upon paraffin embedding. Hemi-brains were then embedded in pairs in paraffin blocks.

To evaluate the precision of section thickness and shrinkage, a caliper was used to measure dimensions of the paraffin block before sectioning was begun. The paraffin block was cut on a microtome (Microm HM340E, Thermo Fisher Scientific, UK) until landmarks of the following coordinates were observed: interaural 4.84 mm; bregma −4.16 mm (just caudal to the disconnection of the tuberoinfundibular stem) (Paxinos and Watson, 1997). The height of the block was then measured with the caliper. In this study, we only used the calculated section thickness to inform about the total volume of the selected brain region.

After re-mounting the block onto the microtome base, a series of adjacent serial sections of 5 μm thickness was cut in the coronal plane using SURS throughout the entire rostro-caudal extent of the SNc. A predetermined sampling fraction was applied (every 50th section), allowing for a sufficient number of section pairs (10–15) to be sampled along the rostrocaudal extent of the SNc. Sections were collected pair-wise onto FLEX IHC slides (DAKO, cat# K802021-2, Agilent technologies, Denmark (DK)) such that two neighboring sections were on each slide. After the last section pair was sampled, the paraffin block was measured again with the caliper and the total number of cut sections was recorded. Thus, the mean section thickness (t) could be calculated by subtraction of the two caliper measurements followed by division by the total number of cut sections (sampled and discarded), see also calculation section below. Slide-mounted sections were dried overnight at 37°C.

### Immunohistochemical staining

Slides were deparaffinized by dipping the slides successively for 15 min in xylene, 2 × 3 min in 99% ethanol, 1 × 2 min in 96% ethanol, 1 × 2 min in 70% ethanol and finally running tap water for 5 min. Sections were subjected to antigen retrieval by immersion of the slides in a TEGTris EGTA pH 9 (TEG) buffer (90°C) for 15 min followed by a TBS buffer wash for 5 min. After antigen retrieval, sections were immunostained using a DAKO automated autostainer (Link 48, Agilent Technologies, DK) according to an optimized protocol. In brief, endogenous peroxidase activity was blocked for 10 min in 1% H_2_O_2_ in TBS + 0.25% Tween-20 (buffer) followed by blocking of unspecific binding by incubation for 20 min in 5% normal swine serum in TBS + 1% BSA + 0.25% Tween-20. Next, the slides were incubated in primary mouse antibody (TH 1:16000, Sigma T2928, lot 110M477, Sigma-Aldrich, DK) diluted in TBS + 1% BSA + 0.25% Tween-20 for 30 min. After a rinse in TBS + 0.25% Tween-20, slides were incubated for 30 min in HRP coupled Envision Polymer rabbit anti-mouse (ready to use DAKO, K4004, Agilent Technologies, DK). After rinsing in buffer, slides were incubated with diaminobenzidine (DAB; 3,3′-diaminobenzidine) 2 component system with chromogen (DAKO, K3468, Agilent technologies, DK). Development was stopped by immersion in water after 10 min. For counterstaining, we used a specialized Mayers Hematoxylin stain (DAKO, Cat# S330930, Agilent technologies, DK), diluted 1:3 in distilled water, with treatment for 1 min. Slides were then dehydrated by brief immersion three times in distilled water, three times in 96% ethanol, 1 × 2 min in 99% ethanol, and 2 × 2 min in xylene, followed by mounted with cover slips using Pertex mounting media (Histolab Products AB, cat#0801). Slides were then dried overnight in a fume head. All slides were scanned on a digital scanner at 20X objective corresponding to 40X magnification (resolution 50,000 pixels per inch, ScanScope AT, Aperio) and saved as Scanscope virtual slides (svs), which is a format compatible with the CAST visiopharm system (Visiopharm, DK) and Image scope software.

### Counting and estimation of tyrosine hydroxylase-positive neurons

**Figure 3:**
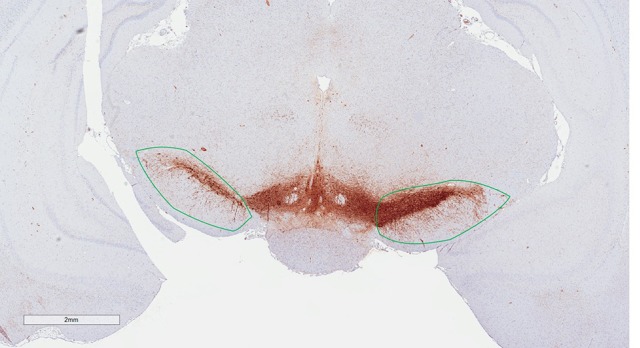
Region of interest (substantia nigra) delineated on a TH immunohistological stained section counterstained with modified Meyer hematoxylin. The section is at the level of bregma −5.6 mm according to the rat atlas of Paxinos and Watson (1997).

**Figure 4:**
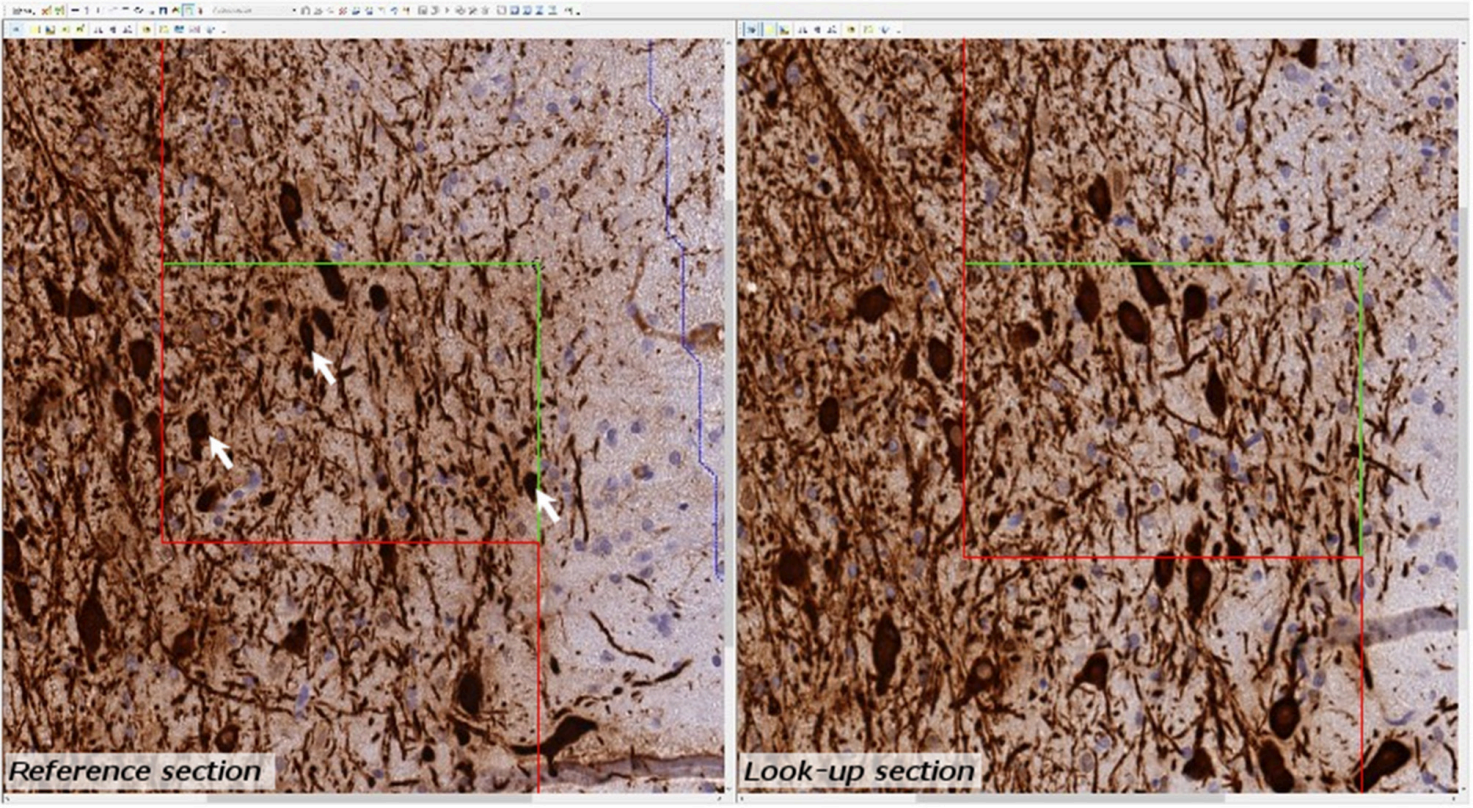
Example of micrograph used for stereological quantification with the physical disector. TH positive neurons (arrow) are counted if they appear in one section (reference section) but not the other (lookup section).

Estimates of total TH-positive neuron numbers in the SNc (separately for left and right hemisphere) were performed using the automated physical disector in a fractionator design on virtual slides. All slide images were loaded onto the CAST Autodisector (Visiopharm, DK) system and an automated alignment and sampling was performed at 10 X magnification. In rare cases where the automatic alignment was not perfect, the researcher performed a manual alignment of the sections. This proved necessary only for sections that were not optimally cut and stained. The region of interest (ROI) was defined on each section using ROI delineation applied to the SNc (see Figure 3). The criterion for delineating the SNc from the ventral tegmental area (VTA) was the localization of the oculomotor nerve root; Whereas the VTA is medial to the root, the SNc is laterally located. SNc was defined extending from anterior-posterior level −5.3 mm with respect to bregma to −6.3 mm according to Paxinos and Watson (1997). The disector counting frame and sampling frequency were adjusted to allow for counting of 150–200 neurons (∑Q^−^) in each of approximately 100 disectors (Figure 4 for counting example).

#### Estimation of the total neuron number using the physical disector, N

Knowing the section sampling fraction (ssf, every 50th sections), the area of the disector counting frame and the relation between the total number of neurons in each subdivision (∑ Q^−^), the total number of TH-positive neurons (N) was estimated as:

$$\begin{array}{l} \text{N}=\left(1/\text{ssf}\times 1/\text{asf}\times \sum {\text{Q}}^{-}\right) \end{array}$$

where asf = a (frame)/X (step) × Y step. The result was divided by two, since the two sections can be compared both ways in a physical fractionator” i.e., with alternating use of the sections in the pair as sampling section and lookup section, respectively” (see Table 1 for calculation examples) (Gundersen, 1986; Dorph-Petersen et al., 2001; Boyce et al., 2010).

**Table 1:** Calculation example for estimation of TH-positive neurons.

**Animal No**	**Region**	**∑Q^−^**	**∑P**	**a(frame) (μm^2^)**	**X-Y step (μm)**	**1/asf**	**1/ssf**	**N**	**V (mm^3^)**
1	SNc control	159	190	60000	387.29	2.50	50	9,938	7.13
2	SNc control	180	181	60000	387.29	2.50	50	11,250	6.79
3	SNc control	172	147	60000	387.29	2.50	50	10,750	5.51

*asf = a(frame)/X × Y step ssf = section sampling fraction, ∑Q^−^ = sum of TH positive neurons, ∑P = sum of points hitting the SNc*.

### Error predictions

Stereology is a reliable method that provides unbiased quantitative results so long as the experimental design meets the necessary critera. In biological sciences there are two primary sources of variation in a dataset: biological coeffecient of variance (CV_biol_) and a sampling variance, which is usually called the coefficient of error (CE) (Gundersen and Jensen, 1987). There will always be a natural variation between animals and a high CV_biol_ can only be compensated by increasing the group size. Sampling variance, on the other hand, can be controlled by the design of the sampling protocol. Thus, CE declines in magnitude if the number of counted objects is increased. In our design, a CE value was considered acceptable if its contribution was less than 50% of the total variance, such that variance due to stereological sampling is less important than the biological variance. In general, a stereological design with 150–200 counting events is usually sufficient to obtain data meeting this criterion for acceptable CE (Gundersen and Jensen, 1987; West, 1999). Knowing the CE is critical for determining whether the sampling protocol can in fact be optimized. This is a crutial point because stereological estimates are non-independent due to the use of systematic uniform random sampling (SURS) in the sampling scheme. This consideration led to the development of a set of equations to calculate the modified CE, which is the sum of two factors, the variance from the point-counting (noise) and the variance from the area sampling (Var_(SURS)_)(Gundersen et al., 1999). The Var_(SURS)_, is the systematic uniformly random sampling variance, which is calculated from:

$$\begin{array}{l} VAR_{SURS}=\;\frac{\sqrt{\left(3\;\left(A-noise\right)-4B+C\right)}}{240}\;where\;\begin{array}{c} A=\;\sum {Q\;}_{i}^{-}{Q\;}_{i}^{-}\;\\ B=\;\sum {Q\;}_{i}^{-}{Q\;}_{i+1}^{-}\\ C=\;\sum {Q\;}_{i}^{-}{Q\;}_{i+2}^{-} \end{array} \end{array}$$

Where noise is, in the case of number estimates, equal to the sum of all cells (∑Q^−^). 240 is a constant appropriate for the current stereological design. The modified CE estimate for volumes include a different noise calculation, which is not presented in this paper.

From the two parameters Noise and VAR_SURS_, ${\text{CE}}_{\sum }{\text{Q}}^{-}$ can be calculated as:

$$CE_{\sum \;Q^{-}}=\;\frac{\sqrt{Noise+\;VAR_{SURS}}}{\sum \;Q^{-}}$$

See Table 2 for calculation example.

**Table 2:** Example of CE calculation.

**CE (N)**	**${\text{Q }}_{\text{i}}^{-}$**	**A:${\text{Q }}_{\text{i}}^{-}$${\text{Q }}_{\text{i}}^{-}$**	**B:${\text{Q }}_{\text{i}}^{-}$${\text{Q }}_{\text{i}+1}^{-}$**	**C:${\text{Q }}_{\text{i}}^{-}$${\text{Q }}_{\text{i}+2}^{-}$**
1	9	81	270	207
2	30	900	690	360
3	23	529	276	322
4	12	144	168	72
5	14	196	84	154
6	6	36	66	90
7	11	121	165	165
8	15	225	225	210
9	15	225	210	105
10	14	196	98	28
11	7	49	14	
12	2	4		
13	0			
14				
sum	158	2,706	2,266	1,713
Noise		158		
Var(SURS)	1.22			
CE (N)=	0.08			

*Q^−^, number of counted TH neurons in a disector section pair*.

When estimating the mean CE for e.g., a treatment group, the following formula is applied

$$\begin{array}{l} \bar{CE}=\sqrt{\frac{CE_{1}^{2}{+CE}_{2}^{2}{\ldots \ldots CE}_{n}^{2}}{n}} \end{array}$$

Where *n* is the number of subjects in the group.

### Statistics

All data were fed into Excel spread sheets and subsequently subjected to relevant statistical analyses using GraphPad Prism (GraphPad Software, La Jolla, CA) or SigmaStat (Systat Software, San Jose, CA), where applicable. Results are presented as mean ± standard error of the mean (S.E.M.). Turning behavior data were analyzed using a two-way repeated measurement analysis of variance (ANOVA). Stereological data were evaluated statistically by an unpaired *t*-test (partial lesion model), or a one-way ANOVA followed by Dunnett's *post-hoc* test (full lesion model). Spearman's correlation test was applied to evaluate the relationship between individual TH neuron number by side and corresponding apomorphine and amphetamine-induced rotations. A *p*-value less than 0.05 was considered statistically significant.

## Results

**Figure 5:**
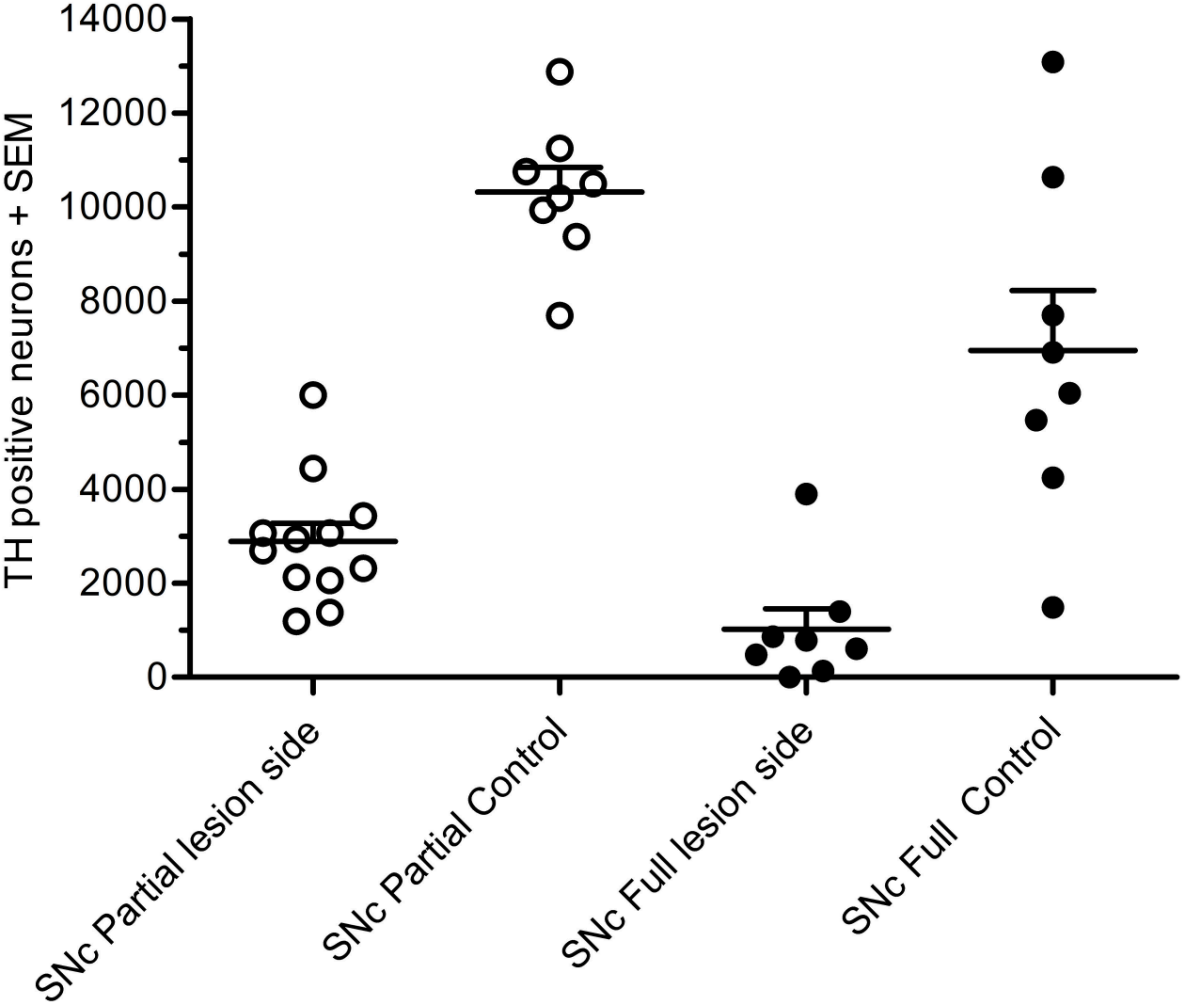
Total number of TH-positive cells in substantial nigra of 6-OHDA-lesioned rats, showing results for the partial lesion model (open symbols) and the full lesion model (filled symbols). Adapted from Hansen et al. (2016), with permission from Brain Research.

**Figure 6:**
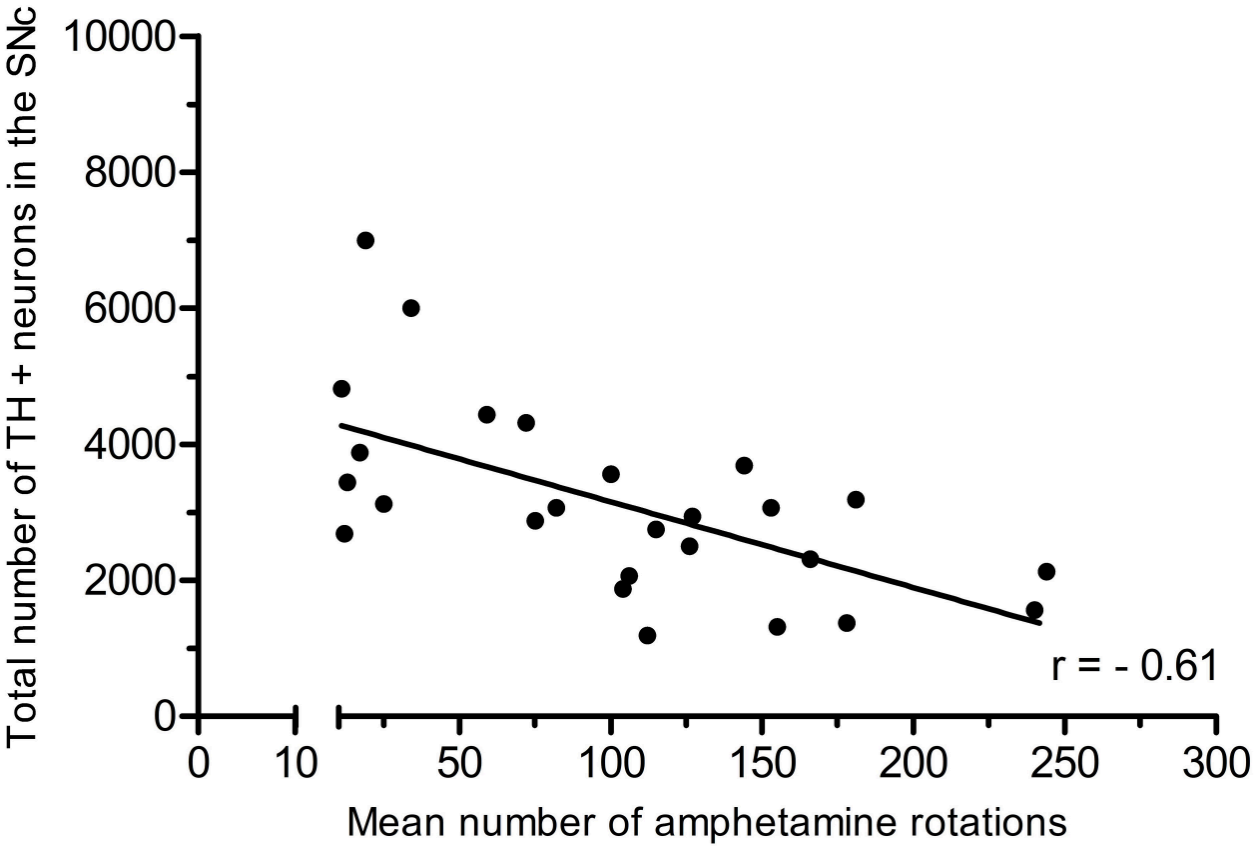
Positive correlation of amphetamine-induced rotations and TH-positive neuron numbers in the SNc. Adapted from Hansen et al. (2016), with permission from Brain Research.

In the partial lesion model, the number of TH-positive neurons in the lesioned (ipsilateral to 6-OHDA infusion) SNc was reduced by 55%, as compared to the contralateral SNc (ipsilateral 4,754 ± 667, mean CE = 0.05; contralateral, 10,321 ± 527, mean CE = 0.31) (Figure 5). Volume estimates showed a reduction in ipislateral SNc volume by 27% (4.80 + 0.32 mm^3^), as compared to the corresponding contralateral SNc volume (6.60 ± 0.27 mm^3^) (data not shown). A correlation analysis of the number of ipislateral nigral TH-positive neurons and corresponding D-amphetamine induced rotations showed a strong inverse linear relationship of mean nigral TH-positive neuron numbers vs. rotation numbers (*n* = 26, spearman *r* = −0.61, *p* = 0.0009, see Figure 6).

In the full nigral lesion model, the number of TH-positive neurons was reduced by 85% (mean ± SEM, ipsilateral 1,020 ± 438, mean CE = 0.09; contralateral: 6,946 ± 1,282, mean CE = 0.44; Figure 5). Comparison with the parital lesion results suggest that there was a 30% reduction in the number of TH-positive neurons on the side contralateral to the repeated 6-OHDA infusion (data not shown). Volume estimates showed a 59% reduction in ipsilateral SNc volume (0.64 ± 0.20 mm^3^), as compared to corresponding contralateral SNc volume (1.58 ± 0.22 mm^3^). There were no significant correlations between apomorphine-induced rotations and TH-positive cell numbers (data not shown). Volume data in this data-set should only be evaluated when compared to the non-infused side.

## Discussion

The physical fractionator method is widely accepted as the current best practice for accurate estimation of total cell numbers in thin tissue sections. This rigorous estimator of object number is based on an unbiased design that minimizes or avoids all known sources of stereological and methodological bias. The primary drawback of the manual fractionator is its low efficiency (low throughput) due to the requirement for manual alignment of pairs of adjacent sections. The automatic method described here allows for an automatic and digital approach to incorporate the unbiased principles of the physical fractionator method. The routine use of digital images in the field of pathology has considerable potential, but has hitherto found scant use. However, this is changing in recent years due to improvement of the technique and wider availability of slide scanners (Taylor, 2011). To minimize the time needed for cell counting, a software system using virtual slides and automated alignment is ideal for physical disector estimation. When using a software-based system, the counting of particles (cells) can be performed off-line from the microscope, and an autodisector on virtual slides combines automatic generation of disector pairs through the use of digital images. When tissue slides are scanned in a digital slide scanner and the autodisector is applied, each slide is partitioned into fields of view, and cells are counted in all fields. Although we have not rigorously compared the time saved with the autodisector system compared to a manual system in the present study of TH-positive neurons, one study by Keller et al. (2013) found that the autodisector on virtual slides was 55–104% faster than the manual physical disector. Although this did not take into account the time expended in scanning, the Aperio scanner used in the present study can process up to 400 microscope slides at a time. Thus, one can initiate scanning in the afternoon, and the process will be completed overnight. In this study, we applied the autodisector on virtual slides, but the autodisector can also be applied directly on tissue slides on a standard microscope equipped for stereology. However, in this event the efficiency will be lower because the image generation is bound to be slower. In addition, acquiring slide images of sufficient quality is time-consuming as a manual process. The use of virtual slides can also be implemented for stereological estimates of volume (as in the present study) and also length and surface areas. However, the time spared by automation of those procedures would probably be less because the time required for microscope stage movement would be almost identical in the two setups. In the present study, the main economy of time was gained by the fast and automatic generation of the disector image pairs. When using virtual slides, it is a matter of concern whether the image quality of the virtual slides is degraded relative to the original tissue slides (Treanor, 2009), and especially important that the plane of focus be acceptable. In general, we found the quality of the virtual slides to be sufficient for evaluation in the stereological setup, but this may depend on the avidity of the particular antibody or staining procedure.

The automated alignment, however, requires optimization of the section sampling technique. Importantly, no cracks or folds should be visible on the sections. Since the digital alignment is based on self-similarity of two image files, high-quality tissue sections are therefore required for obtaining the full benefit of this automatic methodology. In our experience, an experience histologist can undertake manual neuron counting of up to five different ROI per day. However, with a slightly larger initial expenditure of effort, the quality and quantity of the stereological analyses can be improved substantially by applying an automated physical disector principle in a fractionator design for quantitative analysis of immunophenotyped neurons. Compared to optical disectors, the physical disector does not require optimization of shrinkage artifacts, which can be quite challenging for paraffin-embedded material. Further, the use of immunohistologically-stained sections allows for more specific estimations of define cell populations, such as the TH-containing midbrain dopamine neurons estimated in this study. A recent study applied a fully automated optical fractionator design to the counting of NeuN-positive neurons in a mouse neurodegenerative disease model (Mouton et al., 2017), but the present is, to our knowledge, the first study describing the use of the automated physical disector design in a neurodegenerative disease model.

Rats with a partial or full unilateral 6-OHDA nigral lesion showed a 55% or 85% reduction of TH-positive neurons in the ipsilateral SNc, respectively, as compared to the corresponding contralateral SNc, which contained approximately 7000 TH-positive neurons. This is to be compared with estimages of 550,000 pigmented neurons in the human nigra, which is reduced by 66% in patients dying with idiopathic PD (Pakkenberg et al., 1991).

While the severity of motor deficits (amphetamine-induced rotations) in the present study was highly correlated with the extent of DA-ergic neuron loss in rats with a partial nigral lesion, we saw no significant correlation between motor deficits in the apomorphine challenge test and loss of nigral DA-ergic neurons in rats with a more aggressive (“full”) nigral lesion. This could be due to a carry over effect of the 6-OHDA to the contralateral side, leading to a partial sensitization in the “unlesioned” side. Rotational behavior is therefore an imperfect index of the progressive partial nigral 6-OHDA lesion model. On the other hand, the extent of neuronal loss and the proportional rotational responsiveness to a D-amphetamine challenge in the partial nigral 6-OHDA lesion model is in agreement with previous findings using conventional quantification methods of lesion size and rotation (Hefti et al., 1980; Carman et al., 1991; Hudson et al., 1993). However, as the present stereological approach considers the total number of DA-ergic neurons within the complete rostro-caudal extension of the SNc, we contend that it provides an inherently more accurate estimate of DA-containing neurons in the SNc in the partial nigral lesion model, which help to improve the correlation between behavioral and stereological findings. Previous stereological studies of TH-positive cells using an optical disector design in PD models of rodents correspond well with the findings in our study (Deumens et al., 2002; Aponso et al., 2008; Heuer et al., 2013; Boix et al., 2015). We have recently reported the application of this stereological method in the preclinical evaluation of a potential drug therapeutic strategy in PD (Hansen et al., 2016). In addition to neuron counting, the stereological approach can also provide data on volume changes in relevant brain regions. In the context of the present rat 6-OHDA model, partial and full nigral lesions reduced SNc volumes by 27 and 59%, respectively, thus showing that tissue loss being a direct consequence of neuronal loss. This result concurs with volumetric MR-based imaging results in 1-methyl-4 phenyl-1,2,3,6-tetrahydropyridine (MPTP)-treated marmosets (Modo et al., 2017). So long as this relationship holds, tissue volume changes might serve as a surrogate for neuronal loss, but neuron counts remain the gold standard for neurodegenerative disease models.

In conclusion, the automated physical disector provides a useful and effective tool for unbiased estimation of the total loss of nigral dopaminergic neurons in the unilateral 6-OHDA model. This stereological approach should thus prove instrumental in the preclinical evaluation of potential drug therapies for PD, but more generally also potentially extends to the evaluation of neurotoxic or development effects on the number of any cell phenotype ammenable for specific labeling by histology or immunohistochemistry.

## Author contributions

KF: Wrote the manuscript, performed the *in vivo* experiments, performed the stereological work and interpreted the results, PB: Performed the histological and stereological experiments, interpreted the results and co-wrote the manuscript. HH: Revised and supervised the manuscript. JJ: Conceived and supervised the project and created Figure 1.

### Conflict of interest statement

The authors declare that the research was conducted in the absence of any commercial or financial relationships that could be construed as a potential conflict of interest.

## Abbreviations

6-OHDA: 6-hydroxydopamine
asf: area sampling fraction
DA: dopaminergic
hsf: height sampling fraction
i.p.: intraperitoneal
MFB: medial forebrain bundle
PD: Parkinson's disease
ROI: region of interest
s.c.: subcutaneous
SEM: standard error of the mean
SNc: substantia nigra pars compacta
SPD: Sprague-Dawley
ssf: section sampling fraction
SURS: systematic uniform random sampling
TBS: Tris-buffered saline
TH: tyrosine hydroxylase.
